# Mortality and recurrent events after first hospitalisation for heart failure and pulmonary heart disease: a community-based prospective study of 0.5 million Chinese adults

**DOI:** 10.1136/bmjph-2025-003121

**Published:** 2026-09-18

**Authors:** Valirie Ndip Agbor, Yiping Chen, Neil Wright, Christiana Kartsonaki, Jun Lv, Pei Pei, Dianjianyi Sun, Canqing Yu, Liming Li, Zhengming Chen, Wei Jiang, Dan Valle Schmidt, Maxim Barnard, Robert Clarke, Derrick Bennett

**Affiliations:** 1 Nuffield Department of Population Health, University of Oxford, Oxford, England, UK; 2 Peking University School of Public Health Department of Epidemiology and Biostatistics, Beijing, China; 3 Peking University Center for Public Health and Epidemic Preparedness & Response, Beijing, China; 4 Key Laboratory of Major Diseases, (Peking University) Ministry of Education, Beijing, China; 5 NCD Prevention and Control Department, Pengzou CDC, Sichuan, China

**Keywords:** Cardiovascular Diseases, Epidemiological Monitoring, Public Health Practice, Epidemiology

## Abstract

**Background:**

Little is known about inequalities in prognosis of heart failure (HF) and pulmonary heart disease (PHD) in low-income and middle-income countries. The aims of this study were to compare hospitalisation and mortality rates between HF and PHD cases in Chinese adults.

**Methods:**

In a prospective study of 512 724 adults from 10 areas in China Kadoorie Biobank enrolled in 2004–2008, data on first hospitalisations were obtained by linkage to hospitalisation and death registers. Outcomes included 28-day case-fatality ratio (CFR), 5-year cumulative mortality risks (CMR) and 5-year recurrent hospitalisations following first hospital admissions with HF or PHD.

**Results:**

Among first incident hospitalisations with HF (n=7461) and PHD (n=5925), the hospitalisation rates (95% CI) for HF were 125 (122 to 128) and PHD were 99 (96 to 101) per 10^5^ person-years. The hospitalisation rates for HF and PHD were greater in men than women (HF: 132 vs 120; PHD: 123 vs 80) and in rural than urban areas (HF: 130 vs 118; PHD: 165 vs 29). The 28-day CFRs were threefold greater for PHD than for HF (53% (95% CI 52% to 54%) vs 18% (17% to 19%)); the 28-day CFRs were higher for men than women (HF: 22 vs 18; PHD: 58 vs 46). The 5-year CMRs were 47% (35% to 58%) for HF and 70% (59% to 82%) for PHD; the 5-year CMRs were higher in men than women (HF: 55% vs 43%; PHD: 77% vs 62%). The 5-year recurrent hospitalisation risks for HF were 22% (95% CI 20% to 23%) and 34% (32% to 36%) for PHD. The recurrent hospitalisation risks for PHD were also higher in rural than urban areas (36% vs 24%).

**Conclusions:**

Half of all first HF and three-quarters of first PHD cases died within 5 years, highlighting the urgent need to improve treatment for HF and PHD cases in China.

**WHAT IS ALREADY KNOWN ON THIS TOPIC**

- About 50% of first-incident hospitalised cases of heart failure (HF) in Europeans died within 5 years of diagnosis, but little is known about the natural history of HF or pulmonary heart disease (PHD) in East Asians.

**WHAT THIS STUDY ADDS**

- HF cases in East Asians had comparable 5-year mortality rates as in Europeans (47% vs 50%), but the 5-year mortality rates of PHD were much worse than HF (70% vs 47%) in East Asians.

**HOW THIS STUDY MIGHT AFFECT RESEARCH, PRACTICE OR POLICY**

- The 5-year mortality rates for HF (47%) in East Asians were worse than for ischaemic stroke (26%) or myocardial infarction (23%) in East Asians, highlighting the urgent need for improved treatment of HF and PHD in both populations.

## Background

 Heart failure (HF) is a clinical syndrome resulting from structural or functional abnormalities of the left ventricle of the heart. HF affects over 60 million adults worldwide and has a poor prognosis and limited quality of life.^1–5^ About half of all hospitalised cases with HF in Western countries have a recurrent hospital admission with HF within 1 year, and 50% die within 5 years of diagnosis.^2^ The prevalence of HF has increased in recent decades^1^ due to the ageing population, changes in levels of risk factors and improvements in treatment for ischaemic heart disease (IHD).^5^ About 30% of the increase in the global burden of HF cases over the last three decades originated in China.^1^ The 2024 Chinese guidelines for diagnosis and treatment of HF^4^ are consistent with the 2021 European Society of Cardiology (ESC) guidelines^2^ and both advocate measurement of plasma levels of N-terminal pro B-type natriuretic peptide (NT-proBNP) and echocardiographic assessment of left ventricular ejection fraction (EF) to classify HF subtypes.

Pulmonary heart disease (PHD) is also a common cause of hospitalisation in China that shares some presenting symptoms with HF, although it has a very different aetiology.^6,7^ PHD is a clinical syndrome resulting from hypertrophy and dilation of the right ventricle caused by pulmonary parenchymal or pulmonary vascular diseases.^6,7^ The diagnostic criteria for PHD used in China include (1) ECG evidence of pulmonary p wave; (2) ECG evidence of right ventricular hypertrophy and (3) echocardiogram evidence of right ventricular dilatation and right ventricular hypertrophy.^8,9^ The 2018 Chinese Guidelines for diagnosis of PHD advocate clinicians to suspect PHD in the setting of typical symptoms and signs and diagnostic tests consistent with increased pulmonary vascular resistance and pulmonary hypertension (PH).^10,11^ The 2022 ESC/European Respiratory Society guidelines for diagnosis of PH classify PH cases into five groups by their aetiological subtypes: (1) pulmonary arterial hypertension; (2) PH associated with left heart disease; (3) PH associated with lung diseases and/or hypoxia; (4) PH associated with chronic pulmonary artery obstruction and (5) PH associated with unclear or multifactorial mechanisms, although long-term follow-up of cases using this classification are not yet available in either Europe or China.^12^

Much of our knowledge about the natural history of HF has been obtained from follow-up studies of disease registers of HF cases, but large population-based prospective studies provide complementary information on the determinants and outcomes following first hospital admission with HF and PHD.^13–15^ In population-based prospective studies, study participants are typically followed up to record incident cases of HF and PHD by linkage to hospital admission (or death) records, in which cases are diagnosed by local clinicians and coded using the International Statistical Classification of Diseases and Related Health Problems, 10th Edition (ICD-10) codes for HF (I50) and PHD (I27), respectively. In contrast to follow-up studies of disease registers of HF or PHD cases, the results of specialised tests used to classify cases into aetiological subtypes are not routinely available in population studies.^13–15^ However, follow-up of population-based prospective studies^16^ can provide additional evidence on the natural history of HF and PHD in larger numbers of cases and in more diverse settings than in disease registers.

The present report compared the risks of mortality and recurrent events following first hospitalisation for HF and PHD in a 10-year follow-up of the China Kadoorie Biobank (CKB), a population-based prospective study, involving 512 724 adults, recruited from 10 diverse regions in China between 2004 and 2008.^17^ The aims of the present report were to compare the first hospitalisation rates, short-term (28-day case-fatality ratios (CFRs): proportion of deaths from any cause within 28 days due to HF or PHD^16^ and long-term (5-year cumulative mortality risk (CMR)) mortality and 5-year risks of recurrent hospitalisations for HF and PHD, overall and by sociodemographic factors and other comorbidities in the CKB study.^17^

## Methods

### Study design and participants

This report included data from the CKB, a community-based prospective cohort study of 512 724 Chinese adults aged 30–80 years who were recruited during 2004–2008 from 10 geographically diverse areas (five urban and five rural) of China.^17^ The CKB is a collaborative study between the University of Oxford, Peking University and the Chinese Academy of Medical Sciences. Study areas were selected based on population stability, social diversity, differences in levels of established risk factors, major disease burden and the quality of local morbidity and mortality registers.^17^

### Study procedures

The baseline survey was conducted by trained health workers who collected data on self-reported exposures, including socioeconomic and demographic status, lifestyle factors and medical history, using interview-administered electronic-based questionnaires. In addition, physical measures, including blood pressure (BP), were measured using standard procedures and a blood sample was collected for long-term storage.^17^ Participants were considered to have hypertension if they had a measured systolic blood pressure (SBP) of at least 140 mm Hg or a measured diastolic blood pressure (DBP) of at least 90 mm Hg or were receiving treatment for hypertension. The latter was defined as those who reported a diagnosis of hypertension by a physician and use of antihypertensives at baseline.^18,19^ Previously diagnosed diabetes was defined by a ‘yes’ response to the question, “Has a doctor ever told you that you had diabetes?” Among those without previously diagnosed diabetes, diabetes detected by screening was defined if a random plasma glucose level was ≥7.0 mmol/L with time since meal ≥8 hours or ≥11.1 mmol/L with time since last meal of <8 hours or a fasting plasma glucose level of ≥7.0 mmol/L on subsequent testing.^20,21^

### Follow-up for disease outcomes

Study participants were followed-up on a six monthly or annual basis for fatal and non-fatal disease outcomes by electronic linkage via a unique personal identification number, with mortality and morbidity (for stroke, IHD, cancer and diabetes) registries and with the national health insurance (HI) systems that recorded any episodes of hospitalisation. Moreover, active follow-up was undertaken by contacting participants or their relatives on an annual basis for small proportion of individuals (~2%) who were not registered in the HI system. Cause-specific mortality was monitored through China’s Disease Surveillance Points system and electronic HI records (which provide details of all hospital admissions), with annual active confirmation of vital status through local residential and administrative records (which provide vital status and cause-specific mortality statistics for the entire cohort).^22^ The Disease Surveillance Points system provides reliable data on death registration, in which almost all adult deaths were medically certified. For the few deaths (<5%) that occurred without medical attention prior to death, standardised procedures were used to determine probable causes of death by interview of informants (by conducting a verbal autopsy using validated instruments with family members of deceased participants) to record symptoms and signs of deceased participants prior to death. The Disease Surveillance Points system used trained staff to code diseases recorded on death certificates and assign underlying causes using the ICD-10. The ICD-10 codes and procedures for long-term follow-up of incident diseases or death used in this study were comparable with those in other biobank studies in Europe (like UK Biobank) or North America, but their validity for diagnosis of HF and PHD in China has not yet been assessed. For deceased participants, the dates and causes of death (including scanned images of the original death certificates) were reviewed centrally by study clinicians, blinded to any other study information collected at baseline. In analyses of HI records of hospital admissions listing multiple disease outcomes in any episode, the diagnoses of HF and PHD were limited to the first listed causes (ie, main reason) and likewise for death certificates. Previous validation studies involving retrieval and review of medical records from a random sample of cases of major diseases (eg, chronic obstructive pulmonary disease (COPD), myocardial infarction and stroke types) confirmed the reporting and diagnostic accuracy of major diseases in over 90% of cases, but no large studies of validation of reporting accuracy and diagnostic accuracy of HF or PHD have yet been completed in CKB.

### Study outcomes

All fatal and non-fatal diseases were coded using the ICD-10 by trained staff blinded to the baseline information of study participants. First HF and PHD hospitalisation cases were defined as first non-fatal hospital admissions or deaths due to HF (coded as I50) or PHD (I27), respectively. Any first HF and PHD events diagnosed during outpatient consultations were excluded from the present analyses to permit standardised comparisons by age, sex and region within China and between China and other countries. The primary outcomes were (1) first fatal or non-fatal HF and PHD hospitalisation rates; (2) 28-day CFR and 5-year CMR of death from any cause following the first hospitalisation for HF or PHD; and (3) risk of recurrent hospitalisations for HF or PHD after a first non-fatal HF or PHD hospitalisation. The present analyses focused on all-cause mortality rates rather than cause-specific mortality rates to permit comparisons between all-cause mortality rates for HF and PHD occurring in-hospital, or cumulatively by 1 or 5 years after the initial hospital admission. Online supplemental figure S1 shows the number of participants with available data for each analysis.

### Statistical analysis

HF or PHD hospitalisation rates were defined as the number of first hospitalisations per 100 000 person-years of follow-up. Hospitalisation rates across subgroups were adjusted by direct standardisation to the age-at-risk (five groups: 30–49, 50–59, 60–69, 70–79, 80+ years), sex and area (urban and rural) structure of the general CKB population. HF or PHD hospitalisation rates were standardised by age-at-risk (age at disease diagnosis) rather than baseline age.^23^ The hospitalisation rates for HF and PHD were standardised for age, sex and region of the local study population, and the normal approximations to the Poisson rates were used to estimate the 95% CIs for the hospitalisation rates.^24^

The 28-day CFR and 5-year CMR were estimated for participants with first hospitalisation for HF or PHD. The 28-day CFR was the proportion of deaths from any cause among participants with a first HF or PHD hospitalisation occurring within 28 days of diagnosis. In order to estimate the CMR, participants were followed from the date of first HF or PHD hospitalisation to death from any cause. The CMRs (and corresponding 95% CI) were calculated as one minus the estimate of the Kaplan–Meier survival probability (and corresponding 95% CI).^25^ The CFRs and CMRs across subgroups, such as education and number of comorbidities, were adjusted for age at first hospitalisation for HF or PHD, sex and area (urban and rural) by direct standardisation to HF or PHD cases.

Participants were followed up from the date of first non-fatal HF or PHD diagnosis to the date of first recurrent hospitalisation for HF or PHD to estimate the risk of recurrent hospitalisation. Recurrent hospitalisations were defined as any admission for HF or PHD that occurred 28 days after a first diagnosis for non-fatal HF or PHD, and the cumulative incidence function was used to estimate the risks of recurrent hospitalisation, considering death from any cause as a competing event.^26^ The log-minus-log transformation was used to compute the 95% CI for the risk of recurrent hospitalisation.^26^ All time-to-event analyses were censored on 31 December 2018. Online supplemental figure S2 shows the follow-up periods for all time-to-event analyses. The standardised estimates of hospitalisation rates and CFR by subgroups reflect the age–sex–area structure of the subgroups.^24^ The heterogeneity and trends in hospitalisation rates, 28-day CFR and 5-year CMR across subgroups were evaluated using the likelihood ratio test for heterogeneity and trend, respectively. All analyses were performed using R V.4.2.2.

### Public and patient involvement

Prior to enrolment in the study, potentially eligible participants at each of the 10 centres were invited to attend information meetings with representatives of study principal investigators, regional public health leaders and local government officials. These meetings explained the aims and procedures of the study and what participation involved and provided opportunities for questions.

## Results

### Characteristics of the study population

Among the 512 724 included participants, 56 549 (11%) died and 4028 (<1%) were lost to follow-up. Overall, 7461 and 5925 first hospitalisations for HF and PHD were recorded during a median follow-up of 10.1 years from baseline, respectively. The mean age at first hospitalisation was 63.7 (SD 10.3) years for HF and 63.7 (SD 10.3) years for PHD. About 13% (942/7461) of the HF cases also had PHD, and 16% (942/5925) of the PHD cases had HF.

Compared with the overall CKB population, participants with HF or PHD were aged about a decade older, mostly men, had lower levels of education and income, and had a higher prevalence of comorbidities (table 1). PHD cases were mostly rural residents (85.5%), agricultural workers (63.6%) and had a higher prevalence of COPD (50.9%) than the general CKB population. In contrast, cases with HF had a higher prevalence of CVD and Cardiovascular disease (CVD) risk factors, including coronary heart disease (12.5%), hypertension (49.5%) and diabetes (14.5%) than the overall CKB population.

**Table 1:** Baseline characteristics of study participants with first hospitalisation for first HF and PHD

Characteristics	Participants with HF* (n=7461)	Participants with PHD* (n=5925)	All CKB participants (n=512 724)
Age, mean (SD), year	62.6 (9.3)	64.0 (8.4)	52.0 (10.7)
Women, n (%)	55.3	45.3	59.0
Demographic and SES, n (%)			
Rural area	54.6	85.5	55.9
No formal education	31.1	39.8	18.6
Annual household income<10 000 yuan	38.5	59.1	28.2
Agriculture and related work	38.4	63.6	41.7
Lifestyle factors, n (%)			
Ever-regular smokers			
Men	76.5	85.3	74.4
Women	10.7	21.3	3.2
Regular alcohol drinkers			
Men	26.2	23.4	33.3
Women	2.3	4.1	2.1
Physical measurements, mean (SD)			
SBP, mm Hg	142 (23.9)	137 (23.2)	131 (21.3)
DBP, mm Hg	79.5 (12.3)	77.9 (12.0)	77.8 (11.2)
Prevalent conditions, n (%)			
Hypertension	59.5	45.8	35.2
Diabetes	14.5	5.86	5.91
IHD	12.5	4.57	3.02
Stroke and TIA	4.93	2.08	1.73
COPD	19.3	50.9	7.23
At least one comorbidity†	74.5	69.2	44.2
At least one comorbidity (excluding hypertension)	40.8	42.8	17.6

* Participants with HF and PHD are not mutually exclusive.

† Comorbidity was defined as the presence of any of the following: rheumatic heart disease, coronary heart disease, stroke, TIA, hypertension, diabetes, COPD, tuberculosis, emphysema or bronchitis, asthma, cirrhosis, hepatitis B, rheumatoid arthritis, kidney disease or cancer.

CKB, China Kadoorie Biobank; COPD, chronic obstructive pulmonary disease; DBP, diastolic blood pressure; HF, heart failure; IHD, ischaemic heart disease; PHD, pulmonary heart diseases; SBP, systolic blood pressure; SES, socioeconomic status; TIA, transient ischaemic attack.

### Hospitalisation rates for HF and PHD

**Figure 1:**
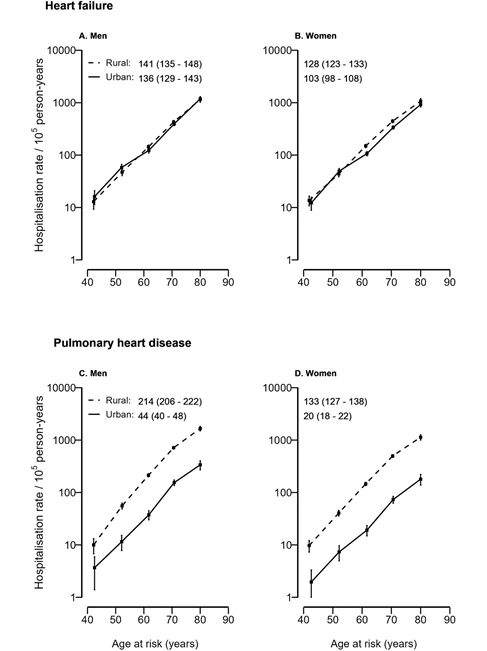
Age-specific hospitalisation rates for heart failure and pulmonary heart disease by sex and area. Age-specific standardised hospitalisation rates were plotted against the mean age-at-risk in each age-at-risk group. The vertical lines through the black squares are the 95% CIs. The y-axis was log-transformed to visualise log-linear trends in hospitalisation rates across age-at-risk groups.

The overall crude hospitalisation rates of HF and PHD were 125 (95% CI 122 to 128) and 99 (96 to 101) per 10^5^ person-years, and the rates increased with age (figure 1). The standardised hospitalisation rates of HF were greater in men (132, 128 to 137) than in women (120, 116 to 123) and in rural (130, 126 to 134) than in urban areas (118, 114 to 122). The standardised hospitalisation rates of PHD were greater in men (123, 119 to 127) than in women (80, 77 to 83) but, importantly, were about sixfold greater in the rural (164, 160 to 169) than in urban areas (30, 28 to 32).

When stratified by sex, the hospitalisation rates of HF were similar in rural and urban men, but were slightly higher in rural than in urban women older than 55 years old (figure 1). By contrast, the hospitalisation rates of PHD were much higher in rural than urban men (214 (206 to 222) vs 133 (127 to 138)) and greater in rural than in urban women (133 (127 to 138) vs 20 (18 to 22)) (figure 1).

**Figure 2:**
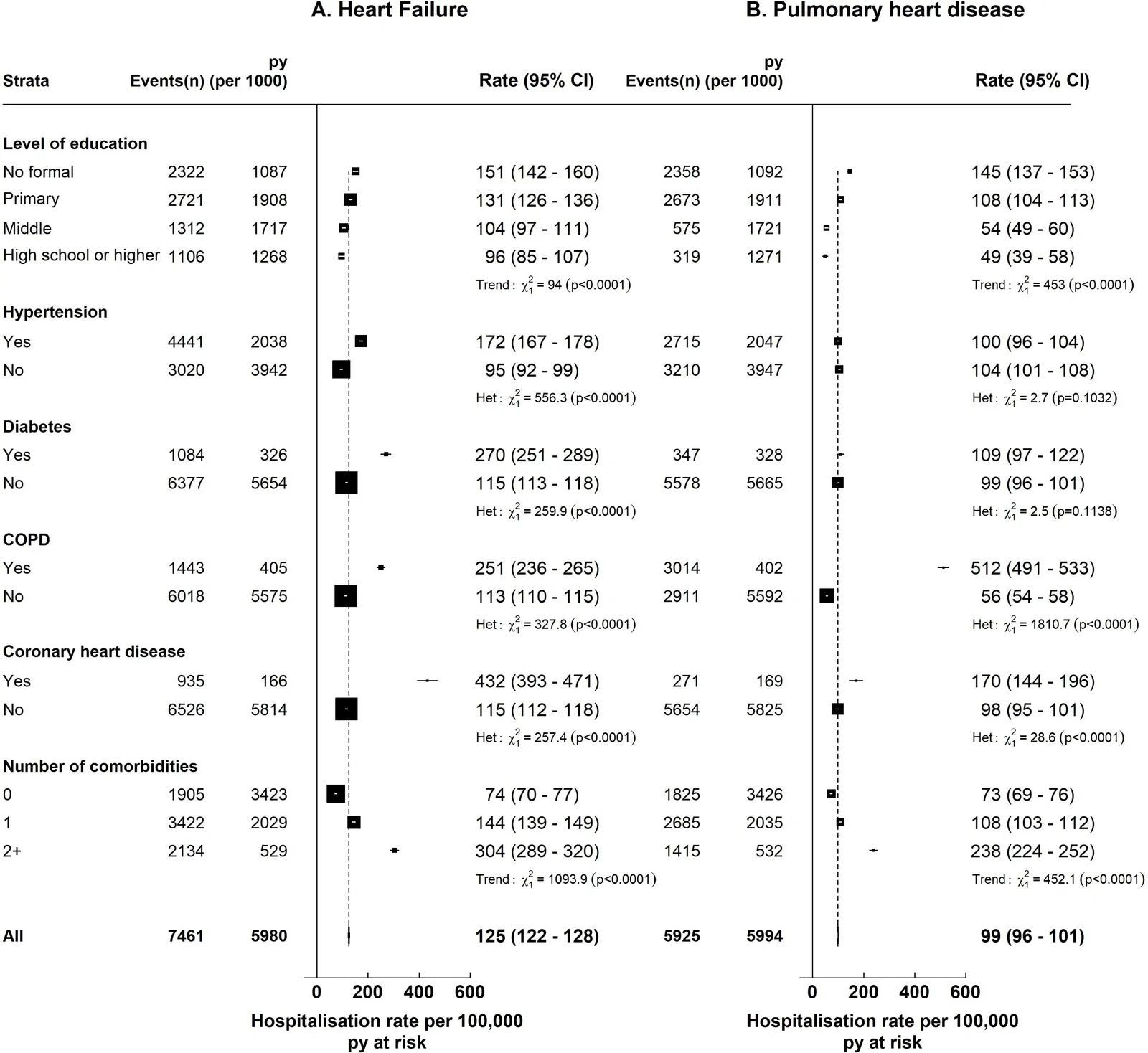
Standardised hospitalisation rates for heart failure and pulmonary heart disease by subgroup. Hospitalisation rates were directly standardised by age-at-risk, sex and area (urban/rural). Comorbidity was defined as the presence of rheumatic heart disease, coronary heart disease, stroke, transient ischaemic attack, hypertension, diabetes, chronic obstructive pulmonary disease, tuberculosis, emphysema, bronchitis, asthma, cirrhosis, hepatitis B, cancer, rheumatoid arthritis or kidney disease.

The standardised hospitalisation rates of HF and PHD were both higher in individuals with lower levels of education, individuals with prevalent COPD, coronary heart disease and presence of comorbidities (figure 2). Individuals with hypertension or diabetes had higher standardised hospitalisation rates of HF (figure 2). The hospitalisation rates of HF and PHD varied substantially by study area (online supplemental figure S3) and were highest in Sichuan (210 per 10^5^ person-years) and lowest in Suzhou (82 per 10^5^ person-years) (online supplemental figure S3). The standardised hospitalisation rates of PHD were highest in Sichuan (377 per 10^5^ person-years) and lowest in Liuzhou (24 per 10^5^ person-years) (online supplemental figure S3). Among all reported cases with ICD-10 codes for PHD, over 70% had associated ICD-10 codes for chronic lung diseases (bronchitis, chronic bronchitis, COPD), but only 7% had codes for concomitant HF. Likewise, among HF cases, over 70% had associated ICD-10 codes for IHD (chronic IHD, angina, myocardial infarction), but only 9% had codes for concomitant PHD.

### 28-day CFR of HF and PHD

**Figure 3:**
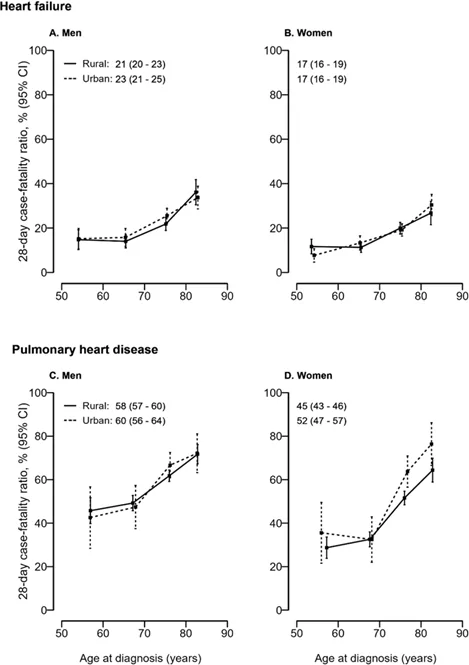
Age-specific 28-day case-fatality ratios (CFRs) after first hospitalisation for heart failure (HF) and pulmonary heart disease (PHD) by sex and area. Age-specific CFRs, represented with black squares, for HF (A) and PHD (PHD, B) were directly standardised by the sex and area structure of China Kadoorie Biobank participants with first incident hospitalisation for HF and PHD, respectively. The vertical error bars through the squares are the 95% CIs. The black squares and vertical error bars in panels (C) and (D) are cumulative mortality risk estimates and their respective 95% CI.

Overall, 1451 and 3128 deaths occurred within 28 days of the first HF (n=7461) and PHD (n=5925) hospitalisation. The overall crude 28-day CFRs of HF were 18% (95% CI 17% to 19%) and of PHD were 53% (52% to 54%). The standardised 28-day CFRs of HF and PHD were higher in men than in women (HF: 22 (20 to 23) vs 18 (16 to 19); PHD: 58 (56 to 60) vs 46 (45 to 49)) with no rural–urban differences (HF: 19 (18 to 20) vs 20 (19 to 21); 52 (51 to 53) vs 56 (53 to 60)). Similarly, sex-stratified analyses showed no rural–urban differences in the 28-day CFRs of HF and PHD in men and in women (figure 3).

The standardised 28-day CFR of HF and PHD did not differ by levels of education, prevalent hypertension, diabetes, stroke or TIA, COPD, number of comorbidities or among cases with comorbid incident PHD or HF, respectively (online supplemental figure S4). By contrast, the 28-day CFR of PHD was higher among those with lower levels of education and those with prevalent hypertension, diabetes, stroke or TIA, COPD and higher numbers of comorbidities at baseline, but was lower in individuals with incident ischaemic heart disease and comorbid incident HF (online supplemental figure S4). The standardised 28-day CFRs of HF varied substantially across CKB areas, with no obvious rural-urban differences (online supplemental figure S3).

### CMR of HF and PHD

**Figure 4:**
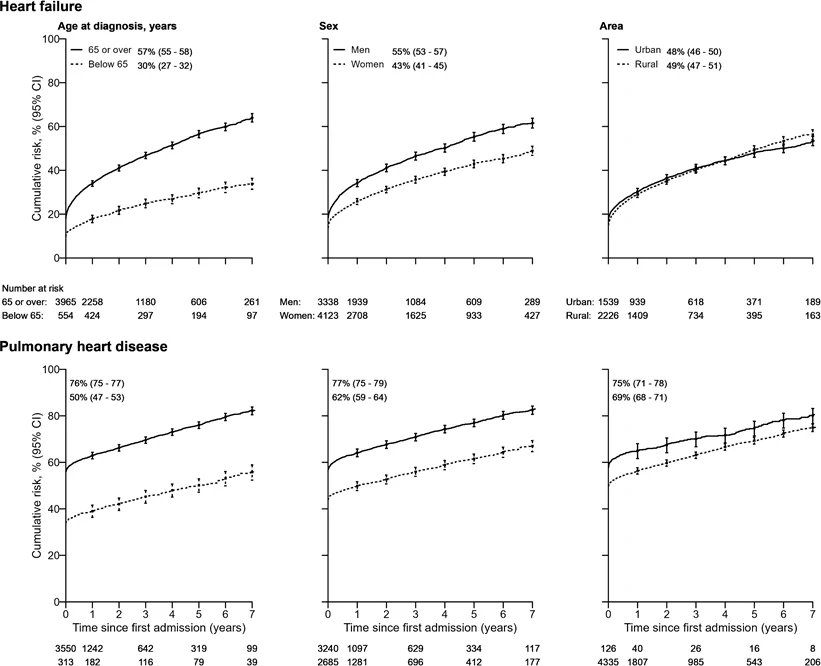
Five-year cumulative mortality risks after a first hospitalisation for heart failure and pulmonary heart disease by age, sex and area. The black squares and vertical error bars represent the point estimates of the cumulative mortality risks and their respective 95% CI. Cumulative mortality curves were directly standardised by age at diagnosis, sex and area as appropriate.

HF and PHD were associated with high 5-year CMRs of 48% (95% CI 47 to 50) and 70% (69% to 71%), respectively (online supplemental table S1). The standardised 5-year CMR of HF and PHD were higher in those aged ≥65 years at diagnosis compared with younger individuals (HF: 57% (55% to 58%) vs 30% (27% to 32%); PHD: 76% (75% to 77%) vs 50% (47% to 53%)) and in men than in women (HF: 55% (53% to 57%) vs 43% (41% to 45%); PHD: 77% (75% to 79%) vs 62% (59% to 64%)) (figure 4). There were no rural–urban differences in the 5-year CMR of HF and PHD (figure 4). The standardised 5-year CMR for both HF and PHD were higher in individuals with lower levels of education and a higher number of comorbidities. Participants with hypertension, diabetes, stroke or TIA and COPD had higher 5-year CMRs. Moreover, the 5-year CMRs of HF and PHD varied substantially by study area (online supplemental figure S3).

### Cumulative risk of HF and PHD rehospitalisation after a first non-fatal event

Overall, 6292 and 2908 first non-fatal HF and PHD hospitalisations were recorded. Among the 6292 cases with non-fatal HF, 912 (14.5%), 274 (4.4%) and 310 (4.9%) had 1, 2 and 3+ recurrent hospitalisations for HF. In contrast, 635 (21.8%), 249 (8.7%) and 330 (11.3%) of the 2908 cases with non-fatal PHD had 1, 2 and 3+ recurrent hospitalisations for the same cause.

The 5-year risks of recurrent hospitalisations were 22% (95% CI 20% to 23%) for HF and 34% (32% to 36%) for PHD (online supplemental table S1). Among participants with HF, the 5-year cumulative risks of recurrent hospitalisation with the same disease were higher among participants aged ≥65 years than those <65 years (26% vs 16%), with no sex or urban–rural differences (online supplemental figure S4). In contrast, the 5-year risks of PHD hospitalisation were higher in rural than in urban areas (36% vs 24%), with no appreciable differences by age and sex (online supplemental figure S4).

## Discussion

This large population-based prospective study of hospitalised cases of HF and PHD in Chinese adults demonstrated high hospitalisation rates of HF and PHD, which increased with age and number of comorbidities and were higher in rural than in urban areas. HF cases had high short-term and long-term mortality risks, and over half of all HF cases died within 5 years of a first hospitalisation. The short-term and long-term mortality risks of PHD were 2.5-fold and 1.5-fold greater than those for HF and 70% died within 5 years. Overall, about 3 in 10 and 1 in 5 HF and PHD cases had repeat hospital admissions for the same disease within 5 years.

Consistent with previous studies,^27–30^ the hospitalisation rates of HF were higher in China than in Western countries. The hospitalisation rates of HF were also higher in older than younger people and those with lower levels of education. Individuals with hypertension, diabetes, prior CVD and those with a higher number of comorbidities also had higher hospitalisation rates of HF. Age was the chief determinant of HF hospitalisation, and hospitalisation rates in those aged 80 years or older were about 70-fold greater than those aged 40 years. With improvements in life expectancy, population expansion and the increasing prevalence of the risk factors of HF worldwide, the findings suggest that there will be a substantial increase in the prevalence of HF in the next few decades without any changes in the population size.^1^

The hospitalisation rates of PHD were higher among individuals with prior COPD and, to a lesser extent, in those with coronary heart disease. In contrast with HF, hospitalisation rates of PHD were unaltered by concomitant diagnoses of hypertension or diabetes, and hospitalisation rates of PHD were chiefly determined by COPD or other chronic lung diseases.

No previous study examined geographic differences in the hospitalisation rates of HF and PHD in China, but the present study demonstrated substantial differences in the hospitalisation rates of PHD and HF within China. These geographic differences in the hospitalisation rates of HF and PHD chiefly reflect differences in the prevalence of risk factors, including socioeconomic, demographic, behavioural and environmental factors.^31^However, the reasons for these geographic differences warrant further investigation in future studies, and this report highlights the need for region-specific approaches for HF and PHD prevention.

Data on the 28-day CFR (short-term mortality) of HF and PHD are limited even in high-income countries,^27,32^ but prospective studies of HF cases in China and other Asian countries have typically been limited in-hospital mortality risks for HF.^29^ The estimates of short-term mortality risks for HF in the present study (18%) were comparable with those reported in a prospective multicentre registry of 4153 acute HF cases in the Czech Republic (20%).^32^ However, the short-term mortality risks for HF in this study were about twofold greater than those in the Framingham Heart Study, involving 1075 HF cases (11%) living in the USA^27^ and also greater than those in a study of 105 399 incident hospitalised HF cases in Malaysia recorded between 2007 and 2016 (11%),^33^ and a pooled estimate from a meta-analysis of 1.5 million chronic and stable HF cases (4.3%).^34^ The 28-day CFR is an important measure of hospital quality of care and predicts long-term disease outcomes.^35^ The CFR varies by calendar year, country and geographic region reflecting differences in public health policies for HF care and differences in access to treatment with evidence-based medicines.

The 5-year CMRs for HF in CKB (47%) were comparable with those of a previous prospective hospital-based multicentre cohort study of 3335 acute HF cases in Beijing, China (55%),^36^ and a meta-analysis of 1.5 million HF cases from high-income countries (43%).^34^ However, comparisons of between-study differences in CMR for HF are complicated by differences in mean age at death, which are typically higher in high-income than in low-income and middle-income countries (approximate mean age: 70 vs 60 years, respectively).^29,37^ Older HF cases also have a higher prevalence of comorbidities, including ischaemic heart disease and atrial fibrillation, which are associated with higher risks of all-cause mortality. Likewise, comparisons of mortality risks between countries are also constrained by differences in the background rates of ischaemic heart disease that are higher in high-income than in low and middle-income populations like China.^1,36^

Importantly, the long-term mortality risks of PHD in this study were about 2.5-fold greater than those in a previously reported prospective study of 4862 cases with COPD and incident HF in the UK.^38^ The differences could reflect disparities in use of treatments for PHD or access to care in both settings. Moreover, PHD cases in CKB typically had a low socioeconomic status, which could also reduce their access to treatment. The high 5-year CMR of about 50% for HF and 75% for PHD was substantially greater than 26% for ischaemic stroke or 23% for acute myocardial infarction reported in the CKB study population.^39,40^ In addition, the 5-year CMR of PHD cases was also substantially higher than those for intracerebral haemorrhage (60%) cases in CKB, which was the stroke subtype with the highest mortality rate in CKB.^39^

The short-term and long-term mortality risks of PHD were about 1.5 to 3-fold fold greater than those for HF, indicating that PHD is a more severe disease with a worse short-term and long-term prognosis than HF. Typically, PHD results from severe chronic respiratory disease (chiefly COPD). In a cohort of Chinese adults admitted with exacerbations of COPD, cases with PHD had more comorbidities, frequent recurrent hospital admissions, longer duration of hospital stay and higher mortality rates than COPD cases without concomitant PHD.^41^

The 5-year risks of recurrent hospitalisation for HF (22%) and PHD (34%) reported in the present study were high, particularly for participants aged over 65 years or those living in rural than in urban areas. Improved treatment is required to prevent recurrent hospitalisations for HF and PHD and reduce the economic burden and premature mortality associated with these diseases.^14,42^ The high risks of recurrent hospitalisations for HF and PHD may also reflect higher rates of comorbidities among HF and PHD cases, as about 70% of CKB participants who developed HF and PHD reported at least one comorbidity at baseline. In addition, the higher risks of recurrent hospitalisations for PHD living in rural than in urban CKB areas may reflect limited access to diagnostic tests or limited access to effective treatments in hospital and during follow-up following discharge from hospital.^43^

This study had several strengths, including being the first study conducted in China to compare the risks of recurrent hospitalisations and mortality rates for both HF and PHD. In addition, this is the first study to explore the long-term risks of mortality and recurrent hospitalisation for PHD in any population. Moreover, the prognosis of cases with HF and PHD was much worse than those for stroke or IHD. However, the study also had several limitations. The findings from this study cannot be generalised to the overall population of China because the CKB is not representative of the Chinese population. The HF and PHD cases in CKB were not adjudicated and, hence, the possibility of some misclassification of these cases cannot be excluded. The CKB study was unable to investigate the prognosis of HF by aetiological subtypes as data on EF and NT-proBNP in hospitalised cases were not collected at the time of this study. Among all reported PHD cases, over 70% had associated ICD-10 codes for chronic lung diseases, but only 7% had concomitant disease diagnoses with ICD-10 codes for HF. Likewise among HF cases, over 70% had associated ICD-10 codes for IHD-related diseases, but only 9% had ICD-10 codes for concomitant PHD. However, the overall diagnostic accuracy of stroke, IHD and COPD cases in CKB was >90–95%,^44–47^ suggesting that Chinese doctors follow worldwide guidelines on diagnosis and we believe that the accuracy of diagnosis of HF and PHD cases in CKB is likely to be comparable with those for other major causes of hospital admissions in China. While it is likely that the positive predictive values for reported diagnoses of HF and PHD will be broadly comparable with those for ischaemic heart disease (ie, myocardial infarction or other IHD), the sensitivity of using reported ICD-10 codes for diagnosis of HF and PHD may be lower in China (since it has a less well-developed primary care service than in Europe or North America). However, such biases should not materially affect the accuracy of within-study comparisons of recurrence rates and short and long-term mortality outcomes between HF and PHD or between HF or PHD and IHD or stroke in the overall population and relevant subgroups.

## Conclusions

Despite some questions about the limited sensitivity of ICD-101 codes to capture all cases of HF and PHD in China, the present 10-year follow-up of >512 724 Chinese adults demonstrated high rates of hospitalisation and mortality and a high frequency of recurrent events following first hospitalisation with HF and PHD in Chinese adults. The findings demonstrated striking differences in the hospitalisation rates of PHD, but comparable rates for HF between urban and rural areas. About half of all first HF and three-quarters of first PHD cases died within 5 years. The findings reinforce the need to improve access to treatment and monitoring in order to reduce the high risks of recurrent hospitalisation and mortality in both HF and PHD.

## Supplementary material

10.1136/bmjph-2025-003121 online supplemental file 1

## Data Availability

Data are available upon reasonable request.
