# Supplementary material for: Mortality and recurrent events after first hospitalisation for heart failure and pulmonary heart disease: a community-based prospective study of 0.5 million Chinese adults
Source: BMJ Public Health. 2026 Sep 18;4(3):e003121. doi: 10.1136/bmjph-2025-003121 (PMC13599994; doi:10.1136/bmjph-2025-003121)
Supplement: Supplementary file 1 [file bmjph-4-3-s001.docx]

**Supplementary material for:**

**Mortality and recurrent events after first hospitalisation for heart failure and pulmonary heart disease: a community-based prospective study of 0.5 million Chinese adults**

**Table of contents**

|  | Page # |
| --- | --- |
| Members of the CKB Collaborative Group | 2-3 |
| **Supplementary Table 1:** Cumulative mortality and repeat hospitalisation risks after heart failure and pulmonary heart disease in CKB | 4 |
| **Supplementary Figure 1:** Numbers of study participants in analyses of different outcomes | 5 |
| **Supplementary Figure 2:** Follow-up period for time-to-event analyses | 6 |
| **Supplementary Figure 3:** First hospitalisation rate, 28-day case fatality, and 5-year cumulative risk of heart failure and pulmonary heart disease, by study area | 7 |
| **Supplementary Figure 4:** Five-year cumulative risk of repeat hospitalisation among survivors admitted for heart failure and pulmonary heart disease, by age, sex and area | 8 |

**Members of the China Kadoorie Biobank Collaborative Group**

***International Steering Committee:*** Junshi Chen^6^, Zhengming Chen (PI)^1,5^, Robert Clarke^1^, Rory Collins^1^, Yu Guo^4^, Liming Li (PI)^5^, Jun Lv^5^, Richard Peto^1^, and Robin Walters^1^.

***International Co-ordinating Centre, Oxford:*** Daniel Avery^1^, Derrick Bennett^1^, Ruth Boxall^1^, Fiona Bragg^1^, Sushila Burgess^1^, Kahung Chan^1^, Yumei Chang^1^, Yiping Chen^1,3^, Zhengming Chen^1^, Robert Clarke^1^, Huaidong Du^1^, Zammy Fairhurst-Hunter^1^, Wei Gan^1^, Simon Gilbert^1^, Alex Hacker^1^, Parisa Hariri^1^, Michael Holmes^1^, Andri Iona^1^, Becky Im^1^, Maria Kakkoura^1^, Christiana Kartsonaki^1,3^, Rene Kerosi^1^, Kuang Lin^1^, Iona Millwood^1^, Qunhua Nie^1^, Alfred Pozaricki^1^, Paul Ryder^1^, Sam Sansome^1^, Dan Schmidt^1^, Rajani Sohoni^1^, Rebecca Stevens^1^, Iain Turnbull^1^, Robin Walters^1^, Lin Wang^1^, Neil Wright^1^, Ling Yang^1^, Xiaoming Yang^1^ and Pang Yao^1^.

***National Co-ordinating Centre, Beijing:*** Yu Guo^2^, Xiao Han^2^, Can Hou^2^, Chao Liu^2^, Jun Lv^3,4^, Pei Pei^2^, Canqing Yu^3^,^4^ and Liming Li^3,4^.

***Regional Co-ordinating Centres:* Qingdao** CDC: Zengchang Pang^7^, Ruqin Gao^7^, Shanpeng Li^7^, Shaojie Wang^7^, Yongmei Liu^7^, Ranran Du^7^, Liang Cheng^7^, Xiaocao Tian^7^, Hua Zhang^7^, Yaoming Zhai^7^, Feng Ning^7^, Xiaohui Sun^7^, Feifei Li^7^. Licang CDC: Silu Lv^8^, Junzheng Wang^8^, Wei Hou^8^. **Heilongjiang** Provincial CDC: Mingyuan Zou^9^, Shichun Yan^9^, Xue Zhou^9^. Nangang CDC: Bo Yu^10^, Yanjie Li^10^, Qinai Xu^10^, Quan Kang^10^, Ziyan Guo^10^. **Hainan** Provincial CDC: Ximin Hu^11^, Jinyan Chen^11^, Xiaohuan Wang^11^. Meilan CDC: Min Weng^12^, Zhendong Guo^12^, Shukuan Wu^12^, Yilei Li^12^, Huimei Li^12^. **Jiangsu** Provincial CDC: Ming Wu^13^, Yonglin Zhou^13^, Jinyi Zhou^13^, Ran Tao^13^, Jie Yang^13^, Jian Su^13^. Suzhou CDC: Fang Liu^14^, Jun Zhang^14^, Yihe Hu^14^, Yan Lu^14^, Liangcai Ma^14^, Aiyu Tang^14^, Yujie Hua^14^, Jianrong Jin^14^, Jingchao Liu^14^. **Guangxi** Provincial CDC: Zhenzhu Tang^15^, Naying Chen^15^, Duo Liu^15^. Liuzhou CDC: Mingqiang Li^16^, Jinhuai Meng^16^, Rong Pan^16^, Qilian Jiang^16^, Jian Lan^16^, Yun Liu^16^, Liuping Wei^16^, Liyuan Zhou^16^, Ningyu Chen^16^, Ping Wang^16^, Fanwen Meng^16^, Yulu Qin^16^, Sisi Wang^16^. **Sichuan** Provincial CDC: Xianping Wu^17^, Ningmei Zhang^17^, Xiaofang Chen^17^, Xunfu Zhong^17^, Jiaqiu Liu^17^, Qiang Sun^17^. Pengzhou CDC: Guojin Luo^18^, Jianguo Li^18^, Xiaofang Chen^18^, Xunfu Zhong^18^, Jiaqiu Liu^18^, Qiang Sun^18^. **Gansu** Provincial CDC: Pengfei Ge^19^, Xiaolan Ren^19^, Caixia Dong^19^. Maiji CDC: Hui Zhang^20^, Enke Mao^20^, Zhongxiao Li^20^, Tao Wang^20^, Xi Zhang^20^. **Henan** Provincial CDC: Ding Zhang^21^, Gang Zhou^21^, Shixian Feng^21^, Liang Chang^21^, Lei Fan^21^. Huixian CDC: Yulian Gao^22^, Tianyou He^22^, Huarong Sun^22^, Pan He^22^, Chen Hu^22^, Xukui Zhang^22^. **Zhejiang** Provincial CDC: Min Yu^23^, Ruying Hu^23^, Hao Wang^23^, Weiwei Gong^23^, Meng Wang^23^. Tongxiang CDC: Chunmei Wang^24^, Xiaoyi Zhang^24^, Kaixu Xie^24^, Lingli Chen^24^, Dongxia Pan^24^, Qijun Gu^24^. **Hunan** Provincial CDC: Yuelong Huang^25^, Biyun Chen^25^, Li Yin^25^, Huilin Liu^25^, Zhongxi Fu^25^, Qiaohua Xu^25^. Liuyang CDC: Xin Xu^26^, Hao Zhang^26^, Huajun Long^26^, and Libo Zhang^26^.

Affiliations:

^1^ Clinical Trial Service Unit and Epidemiological Studies, Nuffield Department of

Population Health, University of Oxford, UK

^2^ Department of Engineering Science, University of Oxford, UK

^3^ Medical Research Council, Population Health Research Unit, University of Oxford, UK

^4^ Chinese Academy of Medical Sciences, Beijing, China

^5^ Department of Epidemiology and Biostatistics, School of Public Health, Peking University

Health Sciences Center, Beijing, China, Peking University Center for Public Health and

Epidemic Preparedness & Response, Beijing, China

^6^ China National Center For Food Safety Risk Assessment, Beijing, China

^7^ Qingdao CDC, Qingdao, Shangdong, China

^8^ Licang CDC, Licang District, Qingdao, Shandong, China

^9^ Heilongjiang CDC, Harbin, Heilongjiang, China

^10^ Nangang CDC, Nangang District, Harbin, Heilongjiang, China

^11^ Hainan CDC, Haikou, Hainan, China

^12^ Meilan CDC, Meilang District, Haikou, Hainan, China

^13^ Jiangsu CDC, Nanjing, Jiangsu, China

^14^ Suzhou CDC, Suzhou, Jiangsu, China

^15^ Guangxi CDC, Nanning, Guangxi, China

^16^ Liuzhou CDC, Liuzhou, Guangxi, China

^17^ Sichuan CDC, Chengdu, Sichuan, China

^18^ Pengzhou CDC, Pengzhou, Sichuan, China

^19^ Gansu CDC, Lanzhou, Gansu, China

^20^ Maiji CDC, Maiji, Tianshui, Gansu, China

^21^ Henan CDC, Zhengzhou, Henan, China

^22^ Huixian CDC, Huixian, Henan, China

^23^ Zhejiang CDC, Hanzhou Zhejiang, China

^24^ Tongxiang CDC, Tongxiang, Zhejiang, China

^25^ Hunan CDC, Changsha, Hunan, China

^26^ Liuyang CDC, Liuya

**Table S1: Cumulative mortality and repeat hospitalisation risks after heart failure and pulmonary heart disease in CKB**

|  | **0** | **1 years** | **2 years** | **3 years** | **4 years** | **5 years** | **6 years** | **7 years** | **8 years** | **9 years** |  |
| --- | --- | --- | --- | --- | --- | --- | --- | --- | --- | --- | --- |
| **All-cause mortality** |  |  |  |  |  |  |  |  |  |  |  |
| **A. Heart failure** |  |  |  |  |  |  |  |  |  |  |  |
| At risk, n | 7461 | 4647 | 3818 | 2709 | 2166 | 1542 | 1055 | 716 | 490 | 316 |  |
| Events, n | 1169 | 998 | 383 | 259 | 155 | 148 | 77 | 59 | 51 | 41 |  |
| Censored, n | 0 | 647 | 446 | 850 | 390 | 474 | 410 | 280 | 175 | 133 |  |
| Cumulative event rate, % (95% CI) | 16% (15–16) | 30% (28–31) | 36% (35–37) | 41% (39–42) | 44% (43–45) | 48% (47–50) | 51% (50–53) | 55% (53–56) | 58% (57–60) | 62% (60–64) |  |
|  |  |  |  |  |  |  |  |  |  |  |  |
| **B. Pulmonary heart disease** |  |  |  |  |  |  |  |  |  |  |  |
| At risk, n | 5925 | 2378 | 2053 | 1325 | 1092 | 746 | 473 | 294 | 199 | 122 |  |
| Events, n | 3017 | 391 | 177 | 149 | 115 | 81 | 64 | 36 | 25 | 25 |  |
| Censored, n | 0 | 139 | 148 | 579 | 118 | 265 | 209 | 143 | 70 | 52 |  |
| Cumulative event rate, % (95% CI) | 51% (50–52) | 58% (56–59) | 61% (60–62) | 64% (63–65) | 67% (66–69) | 70% (69–71) | 73% (72–75) | 76% (74–77) | 78% (76–80) | 81% (79–83) |  |
|  |  |  |  |  |  |  |  |  |  |  |  |
| **Repeat hospitalisation*** |  |  |  |  |  |  |  |  |  |  |  |
| **A. Heart failure** |  |  |  |  |  |  |  |  |  |  |  |
| At risk, n | 6292 | 4080 | 3217 | 2221 | 1728 | 1224 | 827 | 564 | 388 | 242 |  |
| Events, n | 0 | 757 | 246 | 122 | 67 | 36 | 30 | 17 | 9 | 7 |  |
| Death, n | 0 | 861 | 264 | 172 | 101 | 80 | 50 | 32 | 35 | 26 |  |
| Censored, n | 0 | 594 | 353 | 702 | 325 | 388 | 317 | 214 | 132 | 113 |  |
| Cumulative event rate, % (95% CI) | 0% (0–0) | 13% (12–13) | 17% (16–18) | 20% (19–21) | 22% (21–23) | 23% (22–24) | 24% (23–26) | 25% (24–27) | 26% (25–28) | 27% (25–28) |  |
|  |  |  |  |  |  |  |  |  |  |  |  |
| **B. Pulmonary heart disease** |  |  |  |  |  |  |  |  |  |  |  |
| At risk, n | 2908 | 1957 | 1531 | 878 | 692 | 456 | 289 | 181 | 127 | 78 |  |
| Events, n | 0 | 506 | 185 | 102 | 43 | 24 | 19 | 11 | 7 | 1 |  |
| Death, n | 0 | 316 | 103 | 74 | 51 | 40 | 34 | 15 | 12 | 13 |  |
| Censored, n | 0 | 129 | 138 | 477 | 92 | 172 | 114 | 82 | 35 | 35 |  |
| Cumulative event rate, % (95% CI) | 0% (0–0) | 18% (16–19) | 25% (23–26) | 29% (28–31) | 32% (30–34) | 34% (32–36) | 36% (34–38) | 38% (35–40) | 39% (37–41) | 39% (37–42) |  |
| CKB = China Kadoorie Biobank; CI = Confidence interval; n = frequency  *All analyses for repeat hospitalisation excluded all fatal first events | | | | | | | | | | | |

**Study Population**

(N=511,960)

**Study Population**

(N=512,243)

**Short and long-term mortality risk**

(N=7,461 deaths)

**Short and long-term mortality risk**

(N=5,925 deaths)

**5-year repeat hospital admission**

(N=6,292 admissions)

**5-year repeat hospital admission**

(N=2,908 admissions)

**CKB Population**

(N=512,724)

First incident outpatient HF cases

(n=762)

Outpatient PHD

(n=479)

Did not develop HF before 31 December 2018

(n=504,782)

Did not develop PHD

(n=506,318)

Fatal first HF event

(n=1,169)

Fatal first PHD event

(n=3,017)

Missing data (n=2)

**HF**

**PHD**

**Figure S1: Numbers of study participants in analyses of different outcomes.** The analyses were conducted separately for heart failure (HF) and pulmonary heart disease (PHD). Therefore, the number of participants excluded at each stage depended on the trait analysed.

Study censoring date **(31/12/2018)**

Baseline assessment

**(2004–2008)**

Time to first hospitalisation for HF or PHD

Hospitalisation

rate

First

hospitalisation

Time to death from any cause

Cumulative mortality risk

Time to repeat hospitalisation for HF or PHD

First

hospitalisation

Risk of

rehospitalisation

**Figure S2:** **Follow-up period for time-to-event analyses.**

**Figure S3:** **First hospitalisation, 28-day case-fatality and 5-year cumulative mortality risk, by study area.**

**Figure S4:** **Five-year cumulative risk of repeat hospitalisation among survivors admitted for heart failure and pulmonary heart disease, by age, sex and area.**
